# Effectiveness of mepolizumab in severe asthma in Japan: A real‐world study using claims data

**DOI:** 10.1002/clt2.12063

**Published:** 2021-10-06

**Authors:** Hiroyuki Nagase, Jun Tamaoki, Takeo Suzuki, Yasuko Nezu, Masayuki Katsumata, Masaki Komatsubara, George Mu, Shibing Yang, Ashley L. Cole, Rafael Alfonso‐Cristancho

**Affiliations:** ^1^ Division of Respiratory Medicine and Allergology Department of Medicine Teikyo University School of Medicine Tokyo Japan; ^2^ Respiratory Medical Affairs & Development GSK Tokyo Japan; ^3^ Value Evidence & Outcomes GSK Tokyo Japan; ^4^ Value Evidence & Outcomes GSK Collegeville Pennsylvania USA

To the Editor,

1

Patients with severe eosinophilic asthma experience frequent exacerbations and may require oral corticosteroids (OCS) to maintain asthma control.^1^ In randomized controlled trials (RCTs), the anti‐interleukin‐5 monoclonal antibody, mepolizumab, reduces exacerbation rates and OCS use in patients with severe eosinophilic asthma versus placebo.^2^, ^3^ However, due to strict eligibility criteria, RCT populations often have more homogeneous demographics and clinical characteristics than patients treated in real‐world clinical practice.^4^ As such, it is important to validate and complement the results of RCTs with effectiveness data from real‐world settings, which can provide valuable information for clinical decision‐making^5^; these data are currently limited for Japan. The objective of the current study was to evaluate the effectiveness of mepolizumab in reducing exacerbations and OCS use in patients with severe asthma in Japan.

This was a retrospective, observational, self‐controlled study (GSK ID: 213221) of patients from the Japanese Medical Data Center Claims Database (JMDC Inc., Tokyo, Japan)^6^ initiating mepolizumab. The date of the first mepolizumab administration was the index date, and the data were gathered for the 12 months prior to index (baseline period) and up to 12 months following the index date (follow‐up period). Eligible patients for the overall population were ≥12 years of age at index with a diagnosis of asthma during the baseline period and a claim for mepolizumab between June 1, 2016 and August 31, 2018. Additionally, patients had continuous enrollment for the 12 months pre‐ and post‐index. A subgroup analysis of the overall population, which included patients with ≥2 exacerbations during the baseline period and ≥10 mepolizumab administrations during follow‐up, was also performed to assess outcomes in patients with similar characteristics to those in the mepolizumab RCTs.

Outcomes were compared annually/quarterly during baseline and follow‐up and included the annual rate and proportion of patients experiencing an asthma exacerbation (all exacerbations and those requiring hospitalization), the proportion of patients receiving maintenance OCS at baseline (≥5 prednisone‐equivalent mg/day OCS with <15‐day gap at the time of mepolizumab initiation for ≥3 months before index) who discontinued maintenance OCS during follow‐up, changes in median daily OCS dose from mepolizumab initiation (for the 3 months before index date) in patients receiving maintenance OCS at baseline, and the proportion of patients achieving a ≥50% reduction from baseline in daily OCS dose in patients receiving maintenance OCS at baseline. Other outcomes included the number of mepolizumab administrations and discontinuations during follow‐up.

Asthma exacerbations were defined as an asthma outpatient claim and ≥1 prescription for a short course (1–27 days) of systemic corticosteroids (SCS) or an asthma hospitalization with SCS, intravenous aminophylline, or adrenaline recorded during the inpatient stay. For patients currently receiving maintenance therapy (continuous OCS use for ≥180 days with <30 consecutive days gap), asthma exacerbations were defined as requiring an OCS prescription with a mean daily dose at least twice the prior prescription or treatment with intramuscular, or intravenous SCS.

Within the overall population, on‐treatment and on/off‐treatment analyses were performed. For on‐treatment analyses, follow‐up was ended upon mepolizumab discontinuation (>90 days without another mepolizumab injection) or at the first instance of any of the following: 1‐year post‐mepolizumab initiation; claim for another asthma biologic (benralizumab, omalizumab, or dupilumab); or 3 days prior to first bronchial thermoplasty. For on/off‐treatment analyses and the subgroup analysis, mepolizumab discontinuation and reinitiation was permitted, and follow‐up was ended at 1‐year post‐mepolizumab initiation, claim for another asthma biologic, or 3 days prior to first bronchial thermoplasty. Exacerbations per patient‐year during the baseline and follow‐up periods were compared using a generalized estimating equation model with assumed Poisson distributions. Median daily OCS dose was calculated by dividing the total prednisone‐equivalent dose for all days in the quarter‐year by the total number of days for the quarter‐year. Although data from both clinical trial^7^ and real‐world^8^ settings show that mepolizumab improves lung function in patients with severe eosinophilic asthma, the database used in this study does not include lung function parameters.

In total, 61 patients were included in the on‐treatment and on/off‐treatment analyses (two patients in the on‐treatment analysis and two in the on/off treatment analysis discontinued due to first bronchial thermoplasty); of these, 26 patients were included in the subgroup analysis. In the overall population, mean patient age was 52.9 years and just over half (56%) of patients were female. Patients had a mean (standard deviation [SD]) of 286 (106.9) days of follow‐up in the on‐treatment analysis compared with 339 (61.0) and 361 (18.2) days in the on/off‐treatment and subgroup analyses, respectively. During the follow‐up period, patients received a mean (SD) of 9.5 (3.7) mepolizumab administrations in the on‐treatment analysis and 9.9 (3.4) in the on/off‐treatment analysis.

The incidence of any exacerbation was reduced by 43%–56% and exacerbations requiring hospitalization by 58%–100% from the baseline to follow‐up periods (Figure 1A,B), consistent with previous RCTs^3^ and real‐world studies.^9^, ^10^ However, no statistical comparisons were performed, given the absolute number of exacerbations requiring hospitalization. The proportion of patients with an exacerbation was also reduced during the baseline versus follow‐up periods in all analyses (Figure 1C,D).

**FIGURE 1:**
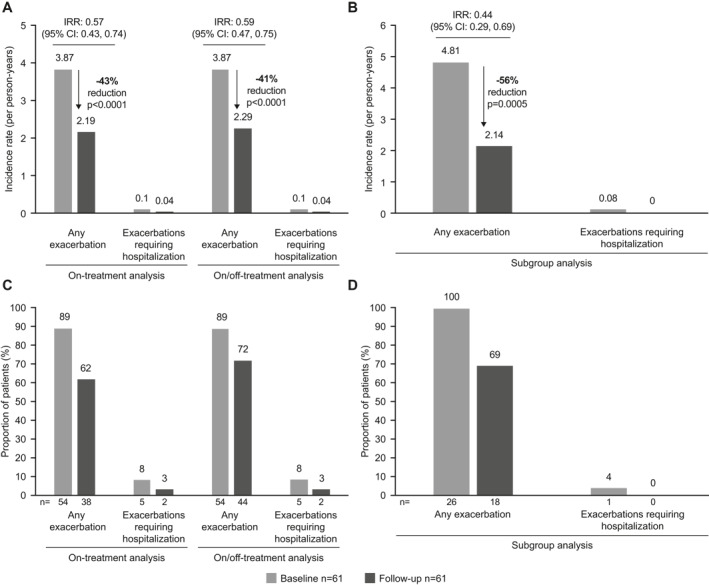
Exacerbation rates in the on‐treatment and on/off‐treatment (A) and subgroup (B) analyses, and the proportion of patients with an exacerbation in the on‐treatment and on/off‐treatment (C) and subgroup (D) analyses. The follow‐up period in the on‐treatment analysis was the time until 1‐year post‐mepolizumab initiation, discontinuation of mepolizumab, or treatment with another biologic or bronchial thermoplasty (whichever is earlier); the follow‐up period in the subgroup analysis was the same as on‐treatment analysis except included only those patients with ≥2 exacerbations during the baseline period and ≥10 mepolizumab administrations during the follow‐up period. CI, confidence interval; IRR, incidence rate ratio

Although the number of events for OCS outcomes was small in the on‐treatment analysis, the proportion of patients receiving OCS maintenance therapy decreased from 26% (*n* = 16) in the 3 months pre‐index to 16% (*n* = 10) in the final quarter of the 12‐month follow‐up period, equating to 6/16 (38%) patients stopping OCS during follow‐up (Figure 2A). The proportion of patients receiving maintenance OCS at baseline who achieved ≥50% reduction in median OCS dose increased throughout the follow‐up period to 60% (*n* = 6/10) in the final quarter, reaching a median (interquartile range) prednisone‐equivalent dose of 3 (1–5) mg/day, a 55% reduction from the baseline dose of 8 (7–11) mg/day (Figure 2B), similar to the 50% OCS reduction in the SIRIUS trial^2^; these reductions are important due to the risk of adverse events and high healthcare costs associated with chronic OCS use.^11^ Results from the on/off‐treatment analysis were consistent with the on‐treatment analysis (Figure 2C,D).

**FIGURE 2:**
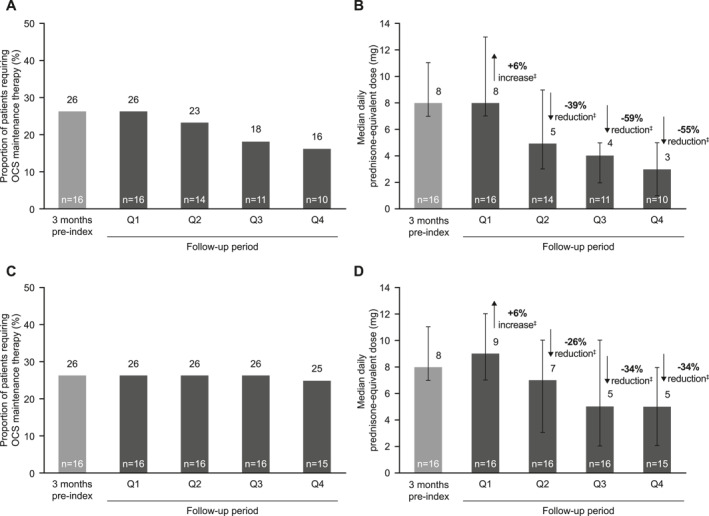
Proportion of patients requiring oral corticosteroid (OCS) maintenance therapy and daily median prednisone‐equivalent dose in the on‐treatment analysis (A, B) and the on/off‐treatment analysis (C, D). For both on‐treatment and on/off‐treatment analyses, follow‐up was ended at the first instance of any of the following: 1‐year post‐mepolizumab initiation, claim for another asthma biologic, or 3 days prior to first bronchial thermoplasty; for the on‐treatment analysis, follow‐up was also ended upon mepolizumab discontinuation (>90 days without another mepolizumab injection); In (B) and (D), the error bars indicate the interquartile range and the *n* numbers at the bottom of the bars indicate the number of patients. ^†^Baseline OCS maintenance was defined as a mean daily dose ≥5 mg/day OCS with <15‐day gap for the 3‐month before index; ^‡^Median percentage reduction from baseline; median of pairwise comparisons: results may differ from calculations made on summary values shown above the bars. OCS, oral corticosteroids; Q, quarter

## CONCLUSION

In conclusion, data from this retrospective database study in patients with asthma in routine clinical practice in Japan demonstrated clinically significant reductions in exacerbations and maintenance OCS use with mepolizumab treatment. These data further support the translation of clinical trial data showing mepolizumab efficacy to real‐world settings.

## CONFLICT OF INTEREST

Hiroyuki Nagase has received speaker fees from AstraZeneca, GSK, Novartis, and Sanofi, has been an advisory board member for GSK, and has received research grants from Boehringer Ingelheim. Jun Tamaoki, Takeo Suzuki, Yasuko Nezu, Masayuki Katsumata, Masaki Komatsubara, George Mu, Shibing Yang, Ashley L. Cole, and Rafael Alfonso‐Cristancho are employees of GSK and hold stocks/shares.

## AUTHOR CONTRIBUTIONS

Hiroyuki Nagase, Jun Tamaoki, Takeo Suzuki, Yasuko Nezu, Masayuki Katsumata, Masaki Komatsubara, George Mu, Shibing Yang, Ashley L. Cole, and Rafael Alfonso‐Cristancho were involved in the conception or design of the study. All authors contributed to the analysis or interpretation of data, drafted the work, or revised it critically for important intellectual content, gave final approval of the version to be published, and agreed to be accountable for all aspects of the work.
